# Very Early Onset of Therapy-Related Acute Myeloid Leukemia with 11q23 Rearrangement Presenting with Unusual PET Findings after R-DA-EPOCH for Primary Mediastinal Large B-Cell Lymphoma

**DOI:** 10.3390/medicina58010048

**Published:** 2021-12-29

**Authors:** Chrysovalantou Chatzidimitriou, Phivi Rondogianni, Maria Arapaki, Athanasios Liaskas, Eleni Plata, Maria K. Angelopoulou, Panagiotis Tsirigotis, Theodoros P. Vassilakopoulos

**Affiliations:** 1 Department of Haematology and Bone Marrow Transplantation, School of Medicine, National and Kapodistrian University of Athens, Laikon General Hospital, 11527 Athens, Greece; cchatzidimitriou@hotmail.com (C.C.); arapaki_m@hotmail.gr (M.A.); ath.liaskas@gmail.com (A.L.); eleniplata@gmail.com (E.P.); mkangelop@gmail.com (M.K.A.); 2 Department of Nuclear Medicine and PET/CT, Evangelismos General Hospital, 10676 Athens, Greece; phrontog@yahoo.gr; 3 Second Department of Internal Medicine, Propaedeutic, School of Medicine, National and Kapodistrian University of Athens, Attikon General Hospital, 12462 Athens, Greece; panagtsirigotis@gmail.com

**Keywords:** primary mediastinal large B-cell lymphoma, R-DA-EPOCH, etoposide, therapy-related, acute myeloid leukemia

## Abstract

Background: R-DA-EPOCH is an effective regimen for PMLBCL, which permits the omission of consolidative radiotherapy in the majority of patients. Patient: We describe a 27-year-old female patient, who achieved a complete remission after treatment with six cycles of R-DA-EPOCH (up to the final level). At 6 months after the end of treatment, PET/CT revealed an unexpected, diffusely increased ^18^FDG uptake by the bone marrow. Simultaneously, pancytopenia with monocytosis was observed. Result: The patient was diagnosed with therapy-related myelodysplastic syndrome, which rapidly evolved into acute myeloid leukemia (t-MDS/AML) with MLL rearrangements. She achieved a complete remission after induction therapy, received an allogenic transplant and remains disease-free 2 years later. Conclusions: The extremely early onset of t-MDS/AML, together with the unexpected PET/CT findings make this case unique and highlights the need for the accurate estimation of the possible dose-dependent risk of t-MDS/AML after R-DA-EPOCH in the real-life setting in patients with PMLBCL.

## 1. Introduction

Primary mediastinal large B-cell lymphoma (PMLBCL) is a rare disease of young adults, with a female predominance [1]. The combination of rituximab, cyclophosphamide, doxorubicin, vincristine and steroids (R-CHOP) has greatly improved the results of CHOP alone [2,3] and has become a reasonable standard of care, usually followed by consolidation radiotherapy. The young age of the patients and the female predominance makes the use of radiotherapy worrisome because of concerns of delayed effects. Although positron emission tomography/computed tomography (PET/CT) may facilitate the omission of radiotherapy after R-CHOP [4,5,6], this remains to be formally addressed by the International Extranodal Lymphoma Study Group (IELSG) randomized trial IELSG-37 [7].

The combination of rituximab with dose-adjusted etoposide, steroids, vincristine, cyclophosphamide and doxorubicin (R-DA-EPOCH) produced impressive results in phase 2 studies [8,9]. Although disease control may be somewhat inferior in real-life case series, R-DA-EPOCH certainly permits the omission of radiotherapy in the overwhelming majority of the patients, thus avoiding potential malignant or non-malignant late toxicity [8,9,10,11,12,13].

R-DA-EPOCH contains topoisomerase II inhibitors, namely etoposide and doxorubicin, and the alkylating agent, cyclophosphamide. Starting from a baseline level (level 1), the doses of these drugs can be escalated according to the patient’s tolerance and disease response up to the sixth or even eighth level [8]. However, these drugs are potentially leukemogenic, as clearly shown in Hodgkin lymphoma after BEACOPP-escalated (bleomycin, etoposide, doxorubicin, cyclophosphamide, vincristine, procarbazine and prednisone) [14,15]. Cases of therapy-related myelodysplastic syndrome and acute myeloid leukemia (t-MDS/AML) are extremely rarely reported after R-DA-EPOCH [8,16].

We describe here an exceptional case of t-MDS/AML after R-DA-EPOCH for PMLBCL with extremely early onset after the end of immunochemotherapy, presented with unexpected findings on follow-up PET/CT, which highlighted the challengedof potential leukemogenicity in this young population.

## 2. Case Report

A 27-year-old female was diagnosed with PMLBCL during the evaluation of a 17 cm mediastinal mass extending to the right lung and the pericardium, a large pericardial effusion and a 5.5 × 4.0 cm spleen lesion. A bone marrow aspiration and biopsy did not reveal lymphomatous infiltration or dysplastic changes. She received six R-DA-EPOCH cycles reaching the highest dose level +6 [8]. More specifically, R-DA-EPOCH level 1 refers to the starting dose level as follows: etoposide 50 mg/m^2^ on days 1–4 (total 200 mg/m^2^), prednisone 60 mg/m^2^ ondays 1–5, vincristine 0.4 mg/m^2^ on days 1–4, cyclophosphamide 750 mg/m^2^ on day 5, and doxorubicin 10 mg/m^2^ on days 1–4 (total 40 mg/m^2^). At each of the following cycles, doses are adjusted by a 20% increase above the last cycle or by a 20% reduction (up to level-2) based on the neutrophil and platelet nadir observed in the previous cycle. Dose adjustments above level +1 apply to etoposide, doxorubicin and cyclophosphamide, and adjustments below level +1 only apply to cyclophosphamide [8]. In the case of uneventful escalation to level +6, as was the case with this patient, the final doses were roughly 124 mg/m^2^ on days 1–4 (total 1986 mg/m^2^) for etoposide in 96 h continuous infusion, 99 mg/m^2^ on days 1–4 (total 397 mg/m^2^) for doxorubicin in 96 h continuous infusion, and 1866 mg/m^2^ on day 5 (total 7447 mg/m^2^) for cyclophosphamide. The clinical course was complicated by pericarditis, treated with non-steroidal, anti-inflammatory drugs (NSAID) and a short course of corticosteroids.

^18^FDG-PET/CT performed 1 and 3 months post-R-DA-EPOCH, without further intervention in the meantime, demonstrated rather mild ^18^FDG uptake interpreted as a Deauville 5-point scale score (D5PSS) of 4 and 3, respectively, with the uptake localized to the 6.6 cm residual mediastinal mass and the pericardium. Although this might indicate active disease, especially after R-CHOP or R-MACOP-B (combination of rituximab, methotrexate, doxorubicin, cyclophosphamide, vincristine, prednisone, bleomycin) [4,5,6], a positive PET/CT (especially D5PSS4) after R-DA-EPOCH does not mandate the use of radiotherapy [8,9].

**Figure 1:**
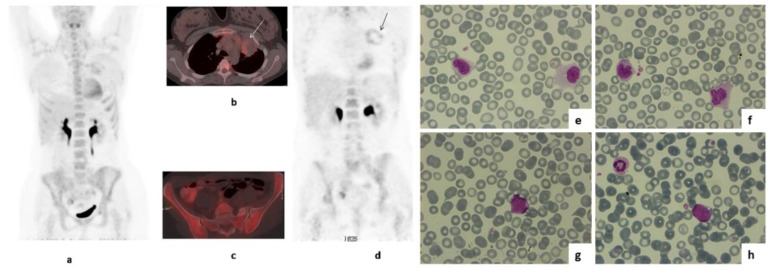
MIP (**a**) axial-fused (**b**,**c**) and coronal PET images (**d**) of ^18^FDG PET/CT performed during the patient’s follow up, 6 months after the completion of treatment with R-DA-EPOCH. There is diffusely increased ^18^FDG uptake by the bone marrow with intensity higher than that of the liver (**a**,**c**,**d**). As no Granulocyte Colony Stimulating Factor (G-CSF) was administered, this was a new finding. Increased peripheral, “ring like” ^18^FDG uptake by the residual large mediastinal mass was also present with intensity higher than that of the liver—-SUVmax_lesion_:4 versus SUVmax_liver_:2,6 (**b** white arrow, **d** black arrow). Peripheral blood smear simultaneously revealed abnormal monocytes (**e**,**f**), and blast cells appeared one month later, when the evolution of t-MDS into acute myeloid leukemia was demonstrated (**g**,**h**).

^18^FDG-PET/CT obtained 6 months after R-DA-EPOCH completion (10.6 months after treatment initiation) revealed an increased, peripheral, “ring-like” ^18^FDG uptake by the residual large mediastinal mass (SUVmax_lesion_: 4.0 versus SUVmax_liver_: 2.6—Figure 1: b, white arrow; d, black arrow), again interpreted as D5PSS4, but with no increase in mediastinal mass size. Unexpectedly, there was a diffusely increased ^18^FDG bone marrow (BM) uptake with an intensity exceeding that of the liver (Figure 1a,c,d) in the absence of recent granulocyte colony stimulating factor (G-CSF) use. Although this pattern of BM uptake was not consistent with BM infiltration by PMLBCL, which would be expected to be multifocal [17,18], further clinical evaluation was recommended.

Blood counts were as follows: Hematocrit 35.3%, hemoglobin 12.3 g/dL, WBC 3.85 × 10^9^/L (neutrophils 0.89 × 10^9^/L, lymphocytes 0.67 × 10^9^/L, monocytes 1.88 × 10^9^/L), platelets 98 × 10^9^/L. Abnormal monocytes (Figure 1e,f) were observed in the peripheral blood smear. Bone marrow aspiration and immunophenotype at that time revealed a population of CD34+CD117+CD13+CD33-HLA-DR+ cells, which consisted of only 1% of CD45+ cells, whereas the percentage of CD64+ cells of the monocytic lineage was 25%, including 12% CD64+CD14− cells. Molecular analysis with reverse transcriptase polymerase chain reaction (RT-PCR, sensitivity of the assay: 10^−4^) and karyotype revealed the presence of Mixed-Lineage Leukemia gene (MLL) rearrangement (t(9;11)(p21;q23);(AF9-MLL)). One month later, the patient progressed to overt t-AML (Figure 1g,h), as a repeat bone marrow biopsy revealed 85% infiltration by blasts expressing HLA-DR, MPO, CD15, PGM-1, CD56, c-kit and CD99 (Figure 1g,h). The 11q23 rearrangement was not detectable, in retrospect, in the pre-R-DA-EPOCH BM sample. The patient achieved a complete remission with the combination of mitoxanthrone, fludarabine, high-dose cytarabine and G-CSF (Nova-FLAG) induction therapy and received one cycle of FLAG consolidation. Subsequently, she was forwarded to a sibling-matched allotransplant and remains in complete remission 32 months later without clinically significant complications.

## 3. Discussion

R-DA-EPOCH is a very effective regimen and obviates the need for radiotherapy in PMLBCL [8,9,10,11,12,13]. High-doses of etoposide, doxorubicin and cyclophosphamide may be complicated by t-MDS/AML. Etoposide and doxorubicin typically cause earlier t-MDS/AML events within 1–5 years from treatment, usually bearing MLL rearrangements, while cyclophosphamide and other alkylating agents cause more delayed t-MDS/AML, carrying complex karyotypes and/or chromosome 5 and 7 abnormalities [1].

The risk of developing t-MDS/AML after R-DA-EPOCH was not thoroughly evaluated. Although R-DA-EPOCH is a popular regimen, especially in the United States, the number of patients that were analyzed in the literature is rather limited and fall within diverse clinical scenarios. In the original R-DA-EPOCH study in PMLBCL, 1/51 patients developed rather late t-AML—at 49 months after the completion of treatment—having reached level +3 [8], while in our Hellenic–Italian–Turkish series, 4/190 cases were recorded (including the case reported here) [19]. These figures were comparable resulting in a rough incidence of approximately 2%. No mention of t-MDS/AML was been made in other real-life series of PMLBCL [10,11,12,20] or other lymphomas [21,22]. In the Intergroup Alliance/Cancer and Leukemia Group B (Alliance/CALGB) randomized trial, 2/241 versus 1/250 patients treated with R-DA-EPOCH and R-CHOP died of t-MDS/AML [16]. However, the median age of these patients was 58 years (>60 years in 43%) and only 30% reached levels 4–6 (14% levels 5–6). In contrast, the median age of patients with PMLBCL in the original R-DA-EPOCH study was 30 years with no patient >60 years old and >50% reached levels 4–6, clearlydue to the much better bone marrow reserves in this young population [8].

This case interestingly illustrates the unexpected PET/CT findings during follow-up of PMLBCL, namely the diffusely increased ^18^FDG bone marrow uptake, due to the development of t-MDS/AML. However, the present case is exceptional due to the extremely short latency period of 6 months from the end or 10.6 months from the start of chemotherapy, respectively. This is the shortest lag-time between the onset of chemotherapy and AML reported so far, along with a single case among those recently reported by Menghrajani et al. [23]. In addition, only 1 of ~80 t-MDS/AML cases developed at 10 months from treatment initiation of Hodgkin lymphoma with BEACOPP, another high-doseetoposide and anthracycline-containing regimen, as inferred from Figure 1 of the published article [24]. The cumulative doses of potentially leukemogenic drugs in fully escalated R-DA-EPOCH for six cycles are 1986 mg/m^2^ for etoposide, 397 mg/m^2^ for doxorubicin, and 7447 mg/m^2^ for cyclophosphamide, in comparison to 3600 mg/m^2^, 210 mg/m^2^,and 7500 mg/m^2^ for BEACOPP-escalated ×6; 4800 mg/m^2^, 280 mg/m^2^, and 10,000 mg/m^2^ for BEACOPP-escalated ×8; and 2400 mg/m^2^, 200 mg/m^2^,and 5200 mg/m^2^ for BEACOPP-baseline ×8. In addition, the BEACOPP variants contain a high cumulative dose of the alkylation agent, procarbazine. All these BEACOPP variants have a well-established leukemogenic potential within large prospective trials, with the risk of t-MDS/AML being <1% [15], 3.2–4.0% [14,15] and 2.2% [14,15] for BEACOPP-escalated x6, BEACOPP-escalated ×8, and BEACOPP-baseline ×8, respectively. The causative relationship between the dose of cytotoxic agents and leukemogenic effect is a major issue, as the efficacy of R-DA-EPOCH is based preciselyon the increase in drug dose according to patienttolerance. Usually, younger patients develop less toxicity and reach higher dose levels, thus are theoretically exposed at greater risk of t-MDS/AML. As the development of t-MDS/AML 6 months after the end of R-DA-EPOCH is extremely unusual, the possibility of a pre-existing lesion in this specific case was suspected and actually excluded by RT-PCR in the pretreatment BM sample.

In a previous study of the German AML Study Group, 54/1897 (2.8%) cases of AML had MLL-rearrangements, more frequently in the group of t-AML (9.4%) compared with de novoAML (2.6%). No case with MLL-rearrangement occurred among AML with a previous myelodysplastic or myeloproliferative neoplasm [24]. The MLL gene is fused with ~50 different partner chromosomal regions, with 9p22 being the most common and more frequently observed in t-AML compared with de novo cases [25]. The recent WHO classification characterizes t(9;11) (AF9/MLL) AML as a distinct entity, differentiating it from the heterogenous group of MLL fusions with various other partner genes.

The short time interval between treatment initiation and t-AML raises the possibility that transformation to AML is the direct result of MLL-rearrangement occurring as a first and single event in a primitive hematopoietic progenitor cell. In accordance with this hypothesis, a previous study showed that concurrent driver gene mutations were extremely rare or absent in 286 patients with t(11q23) AML, with the exception of fms-like tyrosine kinase 3 (FLT3)-activating mutations occurring in 5–10% of cases [26]. The overexpression of ecotropic virus integration site 1 gene (EVI1) was observed in 45.8% of all patients with t(11q23) and was the only factor significantly associated with a poor outcome [26]. In the group of patients with t(9;11), EVI1 deregulation was more frequently observed in t-AML cases and was associated with higher leukocyte counts at presentation, possibly because EVI1 overexpression confers a high proliferative potential to leukemic blasts [26].

A careful observation of the time intervals between treatment initiation and AML onset reveals that patients reported by Menghrajani et al. are divided in two clusters [23], including 12 and 9 patients who developed AML after a median of ~18 months (range, 10.5–22) and ~42 months (range, 38–54), respectively. A better characterization of the two groups may facilitate a greater understanding of the biology of MLL-rearranged AML. EVI1 deregulation may partly explain the very early occurrence in a subset of leukemias as discussed above.

The risk of t-MDS/AML after R-DA-EPOCH should be accurately quantified and correlated to the reached dose level, especially in the young population of patients with PMLBCL. The accurate prediction of prognosis is required in order to tailor treatment to the individualized risk of failure [27,28,29] and potentially restrict more toxic chemotherapy to higher-risk patients. Meanwhile, PET/CT might facilitate the omission of radiotherapy in lower-risk patients [4,5,6,29] in strictly negative patients. Finally, the incorporation of PD-1 inhibitors, which are active in relapsed/refractory disease into the first-line therapy might permit anincrease in efficacy without the toxic effects of more intensive chemotherapy [30,31].
